# Xylooligosaccharides and aerobic training regulate metabolism and behavior in rats with streptozotocin-induced type 1 diabetes

**DOI:** 10.1515/med-2022-0579

**Published:** 2022-10-18

**Authors:** Mariya Choneva, Michaela Shishmanova-Doseva, Ivica Dimov, Krasimir Boyanov, Iliyan Dimitrov, Tatyana Vlaykova, Katerina Georgieva, Petar Hrischev, Anelia Bivolarska

**Affiliations:** Department of Medical Biochemistry, Faculty of Pharmacy, Medical University of Plovdiv, 15 A, Vassil Aprilov Blvd., Plovdiv, 4002, Bulgaria; Department of Pharmacology, Toxicology and Pharmacotherapy, Faculty of Pharmacy, Medical University of Plovdiv, 15 A, Vassil Aprilov Blvd., Plovdiv, 4002, Bulgaria; Department of Physiology, Faculty of Pharmacy, Medical University of Plovdiv, 15 A, Vassil Aprilov Blvd., Plovdiv, 4002, Bulgaria

**Keywords:** cognition, gut–brain-axis, gut microbiota, oligosaccharides, type 1 diabetes mellitus

## Abstract

Type 1 diabetes mellitus is characterized with decreased microbial diversity. Gut microbiota is essential for the normal physiological functioning of many organs, especially the brain. Prebiotics are selectively fermentable oligosaccharides [xylooligosaccharides (XOS), galactooligosaccharides, etc.] that promote the growth and activity of gut microbes and influence the gut–brain axis. Aerobic exercise is a non-pharmacological approach for the control of diabetes and could improve cognitive functions. The potential beneficial effect of XOS and/or aerobic training on cognition, the lipid profile and oxidative stress markers of experimental rats were evaluated in this study. Male Wistar rats were randomly divided into three streptozotocin-induced diabetic groups and a control group. Some of the rats, either on a XOS treatment or a standard diet, underwent aerobic training. The results showed that the aerobic training independently lowered the total cholesterol levels compared to the sedentary diabetic rats (*p* = 0.032), while XOS lowers the malondialdehyde levels in the trained diabetic rats (*p* = 0.034). What is more the exercise, independently or in combination with XOS beneficially affected all parameters of the behavioral tests. We conclude that aerobic exercises alone or in a combination with the prebiotic XOS could ameliorate the dyslipidemia, oxidative stress, and cognitive abilities in experimental type 1 diabetic animals.

## 1 Introduction

Type 1 diabetes mellitus (T1DM) is an autoimmune disease that results from an immune-mediated destruction of the beta (β) cells of the pancreatic islands, producing insulin [1]. The exact factors, responsible for the induction of T1DM, have not been discovered yet; however, genetic predisposition in combination with environmental factors such as viral infections, antibiotic treatment, consumption of proteins of foreign origin, lack of breastfeeding, etc., are found to contribute to the development of T1DM [2]. A considerable number of studies have proven the relation between the gastrointestinal microbiome and the development and severity of diabetes mellitus (DM). The most common cases of dysbiosis in T1DM include deficiency of short-chain fatty acid (SCFA)-producing bacteria and increased number of *Bacteroidetes* sp., which is associated with a decreased microbial diversity and gut integrity [3].

An increasing number of studies suggest that T1DM is associated with various forms of cognitive impairment [4,5]. Dyslipidemia, which is common in T1DM [6], is proven to influence cognition and memory as hypertriglyceridemia and hypercholesterolemia are found to cause cognitive damage [7,8]. An increased production of reactive oxygen species, present in T1DM, is also a factor, contributing to the cerebral dysfunctioning [9].

Emerging data have demonstrated that commensal microbiota could have an impact on stress response, cognitive function, and motor and communicative abilities and it can also result in mood alterations, such as anxiety- and depressive-like symptoms [10,11,12]. The connection between the intestinal microbes and the brain is known as gut–brain axis [13]. In this respect, gut microflora is essential not only for gut health but also for normal physiological functioning of other organs, especially the brain and its regulation could be used as a potential treatment of different neurological disorders [14].

Prebiotics are selectively fermentable oligosaccharides [xylooligosaccharides (XOS), galactooligosaccharides, fructooligosaccharides, etc.], occurring naturally in high-fiber foods. They render beneficial effects in the organism by promoting the growth and activity of gut microbes and they also influence the gut–brain axis and are linked to human health [15]. XOS are a mixture of oligosaccharides containing xylose residues linked by β-1,4 bonds [16]. It has been demonstrated that XOS mitigate oxidative stress, inflammation, hyperglycemia, and hyperlipidemia through decreasing the levels of low-density lipoproteins and total cholesterol and increasing the levels of high-density lipoproteins (HDL) [17,18]. Only few recent studies have revealed that some functional oligosaccharides or prebiotic mixtures could influence positively the gut–brain axis and improve cognitive dysfunction and locomotor behavior through influencing some of the mentioned mechanisms [19,20,21]. However, the information about the effect of XOS on cognitive and mood abilities is still very scarce.

Physical training has been demonstrated as a non-pharmacological approach for the control of DM and its complications through ameliorating the physiological and psychological conditions of diabetic patients [22]. Physical exercise exerts a beneficial impact on body weight and abnormal metabolic function and improves muscle strength, respiratory function, heart rate, and blood flow [23,24]. Moreover, aerobic training could improve cognitive functions and influence positively some psychiatric co-morbidities, such as anxiety and depressive-like symptoms in patients with DM [25]. In this sense, physical training provides mental-health benefits and improves the quality of life of this population [24].

Taken together all these data, in the current study, we aimed to evaluate the impact of aerobic training and XOS supplementation on the lipid profile, oxidative stress markers, cognitive capacity and anxiety of experimental rats with streptozotocin (STZ)-induced T1DM.

## 2 Materials and methods

### 2.1 Animals

Thirty-one 8-week-old male Wistar rats with the weight of 195 ± 30 g were sourced from the vivarium of the Medical University of Plovdiv. The rats were randomly divided into four groups (*n* = 7–8): (1) Sed-STZ – sedentary diabetic rats on a standard diet; (2) Ex-STZ-XOS – trained diabetic rats, fed with a XOS supplement; (3) Ex-STZ – trained diabetic rats on a standard diet; and (4) Sed-veh – sedentary healthy rats on a standard diet (control group).

Five animals were housed per cage under standard conditions – living space of 350 cm^2^, humidity of 55 ± 10%, temperature of 22 ± 2°C, with a 12-hour light/dark cycle. The animals had free access to food and water.

The rats from the Sed-STZ, Ex-STZ-XOS, and Ex-STZ groups received an intraperitoneal (i.p.) STZ injection in a dose of 60 mg/kg body weight for the induction of type 1 diabetes. The animals did not receive any antihyperglycemic treatment. The rest of the animals (the control group) were injected with the same volume of saline.

One week after the STZ administration, the animals from the Ex-SZT-XOS group received a XOS supplement every day for 8 weeks in the dose of 100 mg/kg. The XOS was diluted in distilled water and given *per os*. The XOS used in the experiment with a trade name – XOS powder was sourced from Lenzing AG, Lenzing, Austria. The distribution of the XOS in the product was as follows: 13% with a degree of polymerization (DP) of 2, 19% with a DP of 3, 11% with a DP of 4 and 60% with a DP of 5 and more.

**Figure 1:**
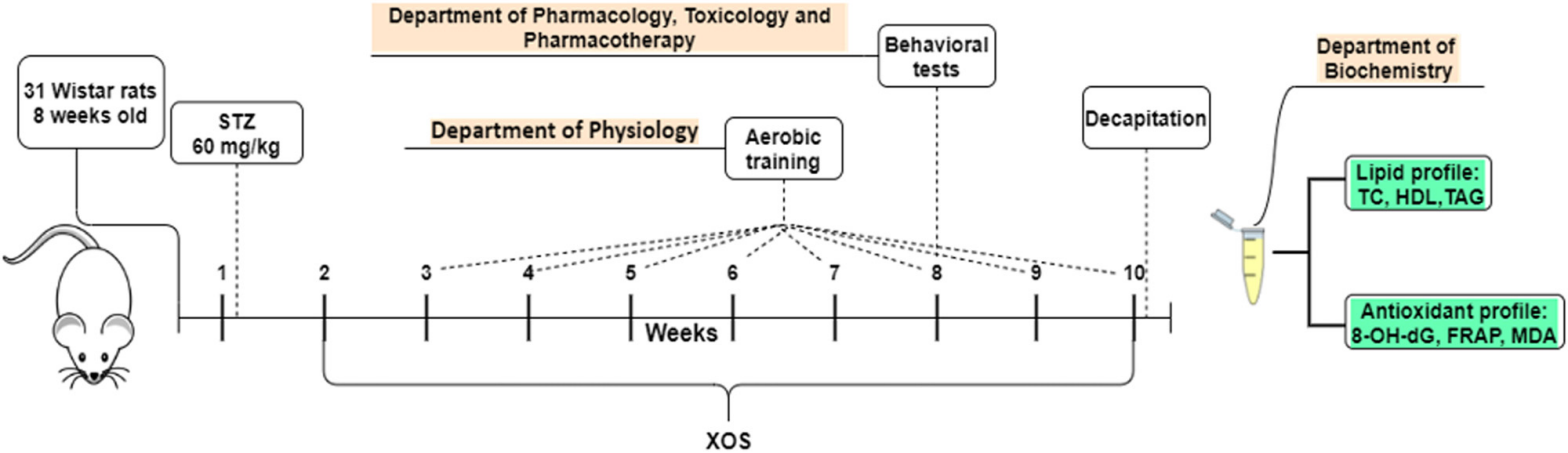
Timeline of the experiment. TC – total cholesterol; TAG – triacylglycerols; 8-OH-dG – 8-hydroxy-2-deoxyguanosine; FRAP – ferric-reducing ability of plasma; MDA – malondialdehyde.

The rats were subjected under these conditions for 10 weeks. The schedule of the experiment is given in Figure 1. At the end of the tenth week, the animals were fasted overnight and decapitated after a treatment with an overdose of the anesthetic ketamine/xylazine (87.5/12.5 mg/kg). Blood serum was collected from the hearth right after the decapitation and was immediately frozen at −18°C.

All experiments in this study were approved by the Medical University of Plovdiv (resolution of the University Ethic Committee No. 2/13.06.2019) and were performed in compliance with protocols approved by the Bulgarian Agency for Food Safety (BAFS resolution No. 150/09.04.2019) and comply with the ethical standards of.

### 2.2 Diabetic model

The diabetic state was induced by the injection of a single dose of STZ in the area of the abdomen. The STZ solution was prepared in a citrate buffer, pH 4.5, in accordance with the method of Furman [26].

The substances used for the buffer were:

• 5.78 g citric acid × 1 H_2_O (*M*_w_ = 210.14 g/mol) dissolved in 50 mL of distilled water and

• 0.71 g Na_2_HPO_4_ (*M*_w_ = 141.96 g/mol) dissolved in 50 mL distilled water.

In order for the wanted pH of the buffer to be reached, 10 mL of the citric acid solution and 45–50 mL of the Na_2_HPO_4_ solution were used. A stock solution with a concentration of 30 mg/mL was prepared (1 g of dry STZ substance was dissolved in 33.3 mL of buffer) and a dose of 60 mg/kg body weight was applied to each of the experimental animals in the STZ groups [27].

### 2.3 Aerobic training

The rats from the Ex-SZT-XOS and Ex-STZ groups were trained on a treadmill for small experimental animals EXER-3R-Treadmill (Columbus Instruments, Columbus, OH, USA) with a band speed of 16 m/min and slope of 5° (about 55–60% of VO_2max_), 5 days a week for 8 weeks. The duration of the training was 20 min on the first day and it was gradually increased with 5 min every other day. At the end of the second week, the duration of the training reached 40 min and it was kept until the end of the experiment. The exercise intensity was set below the maximal steady state established for Wistar rats during treadmill running [28], indicating that the training is aerobic. The rats from the other groups were sedentary.

### 2.4 Behavioral tests

#### 2.4.1 Activity cage

The activity cage test was conducted with an apparatus that included a transparent plastic square box (Biological Research Apparatus, UgoBasile, Italy) with a size of 40 cm. The number of two different types of movements, horizontal and vertical, was recorded automatically by a printer. The movements were detected by infrared sensor array that was located at both sides of the cage. Each session lasted 180 s and was conducted under identical conditions. The animals were all tracked individually. The test was conducted for the spontaneous locomotor activity to be measured.

#### 2.4.2 Passive-avoidance step-down test

The rats were placed individually in an automatic step-down device (UgoBasile, Italy) for passive avoidance with negative reinforcement, as previously reported. A test chamber (14 cm × 33 cm) was used equipped with a vibrating plastic platform (14 cm × 19 cm) and the rats were placed on it at the beginning of the experiment. They were trained twice with a 60-min interval between the sessions. The reaction latency was measured when the rats attempted to climb down from the platform with three or all four paws and, at the same time, they were given an electric foot shock (0.4 mA for 10 s) through the grid. The learning session was conducted in 1 day and consisted of three trials with a 30 min interval between them. Three hours after the last trial, the test for short-term memory was conducted, while the test for long-term memory was performed 24 h later. The reaction latency (remaining on the platform for 60 s) was considered a measure of learning and retention.

#### 2.4.3 Elevated plus maze test

The apparatus for the elevated plus maze test included two open arms (50 cm^2^ × 10 cm^2^), two enclosed arms (50 cm^3^ × 10 × 50 cm^3^), and a central platform (10 cm^2^ × 10 cm^2^) that was raised 50 cm from the ground. The experimental animals were initially placed in the center of the maze, facing forward an open arm, and were given 5 min to explore it. The parameters that were calculated included: (1) the number of entries in the open arms; (2) the number of entries in the enclosed arms; (3) the time (s) spent in the open arms; and (4) anxiety index = 1 − [(open arms time/total time) + (number of entries in open arms/total number of entries)/2]. An ethanol solution (60% volume) was used for the disinfection of the alleys of the maze after the removal of each rat.

#### 2.4.4 Y-maze

The spatial working memory of the rats was assessed using a Y-maze alternation task consisting of three black, opaque plastic arms at a 120° angle from each other. Each rat was placed at the end of one arm and allowed to move freely through the maze for 5 min. The series of arm enters was recorded visually. Alternations were defined as the rat entered different arms of the maze three times in succession, driven by the curiosity to explore previously unvisited areas. An entry occurs when all four limbs are within the arm.

The number of triads was defined as the number of alternations (e.g., ABC, BCA, or CAB, but not ABA). The percentage of spontaneous alternations was calculated by dividing the total number of alternations by the total number of entries minus 2. Therefore, the percentage of alternations is used as an index of spatial working memory.

#### 2.4.5 Object recognition test (ORT)

The ORT included three separate phases: a training session or a first trial (T1) and a training test interval and a test session or a second trial (T2). The apparatus the test was conducted with was an open field (OF) (50 cm^3^ × 50 cm^3^ × 50 cm^3^). Glass or plastic objects were used in the testing. The rats were adjusted to the OF for 15 min on the first day. Twenty-four hours later, the test was performed. During T1, each animal was situated into the arena and two identical objects (A1 and A2) were placed in front of them for 5 min. The objects were placed about 10 cm away from the wall in a symmetrical position. The rats were then returned to their home cage for an inter-trial interval that lasted 60 min. The apparatus and the objects were cleaned with 70% ethanol solution after each rat. After T1 and the interval, one of the objects was replaced with a novel one (B). In T2, one familiar object (A1) and the novel object (B) were exposed to the rats for 5 min. An exploration of the objects was considered when: the animal directed its nose toward the object at no more than 2 cm and/or touched it with its whiskers/nose and licked it. If the rats sat on the object that was not viewed as exploratory behavior. The time of exploration (s) of each of the objects in the separate trials was detected and the listed parameters were measured: the total time each animal spent exploring the two objects in T1, and the discrimination index (DI), calculated by the following equation: (*T*_B_ − *T*_A_)/(*T*_B_ + *T*_A_), where *T*_B_ is the exploration time of the novel object, *T*_A_ is the exploration time of the familiar object, and *T*_B_ + *T*_A_ is the total time spent exploring the two objects.

### 2.5 Assays for the lipid profile and oxidative stress markers of the rats

The quantitative analyses were performed by an enzyme-linked immunosorbent assay (ELISA) microplate reader HumanReader.HS, HUMAN (Wiesbaden, Germany). Commercially available kits were used for the determination of the serum total cholesterol (TC) levels [Total cholesterol (Rat) ELISA kit, MyBioSource Inc., San Diego, CA, USA], HDL levels [Rat HDL (High Density Lipoprotein) ELISA Kit, Elabscience Biotechnology Inc.], triacylglycerol (TAG) levels [Triglyceride (Rat) ELISA Kit, BioVision Inc., Milpitas, CA, USA], 8-hydroxy-2-deoxyguanosine (8-OHdG) levels [8-OH-dG (8-Hydroxydeoxyguanosine) ELISA Kit, Elabscience Biotechnology Inc.], and MDA levels [Rat MDA (Malonaldehyde) ELISA Kit, Wuhan Fine Biological Technology Co., Ltd.].

FRAP (ferric-reducing antioxidant power) was assessed by the method described by Benzie and Strain [29] with certain modifications. The FRAP reagent contained 100 mL 300 mmol/L acetate buffer, pH 3.6 (3.1 g CH_3_COONa × 3H_2_O), 10 mL 20 mmol/L FeCl_3_ × 6H_2_O, and 10 mL 10 mmol/L TPTZ (2,4,6-tripyridyl-*s*-triazine, Sigma-Aldrich, USA). A volume of 2.85 mL of the reagent was mixed with 0.15 mL serum, the mixture was incubated for 30 min at 37°C in the dark, and the absorption was measured at 593 nm. The results were expressed in micromoles Trolox equivalent.

### 2.6 Statistical analysis

The results are reported as mean ± standard error of mean (SEM). One-way analysis of variance (ANOVA) followed by Tuckey’s *post hoc* test was used for assessing the significance in the differences between three or more independent groups. Two-way multivariate ANOVA was used for the evaluation of the effect of diabetes and aerobic training on the parameters from the behavioral tests. The statistical analyses were performed with the statistical program SPSS, version 17.0 (SPSS Inc., Chicago, IL, USA).

## 3 Results

### 3.1 Behavioral tests

#### 3.1.1 Activity cage

**Figure 2:**
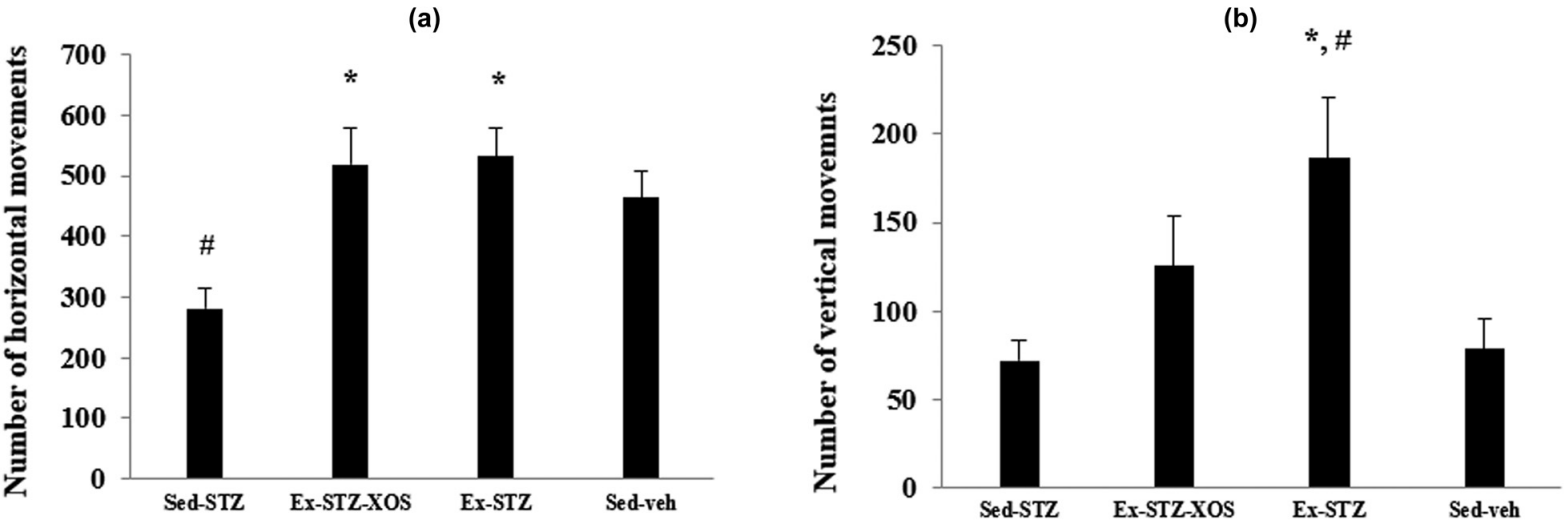
Effect of XOS and/or aerobic training on the results from the activity cage test. (a) Horizontal activity; (b) Vertical activity. ^*^Significant differences in comparison to Sed-STZ. ^#^Significant differences in comparison to Sed-veh.

There was a significant main effect of the diabetes [*F*(1, 33) = 7.716, *p* < 0.01] and exercise [*F*(1, 33) = 18.454, *p* < 0.001] on the number of the horizontal movements. The *post hoc* test revealed that the Sed-SZT group had a significantly lower number of movements in comparison to the Sed-veh animals (*p* = 0.047). Both Ex-SZT and Ex-STZ-XOS groups demonstrated increased locomotor activity compared to the Sed-SZT group (*p* = 0.004 and *p* = 0.006, respectively) (Figure 2a).

The training program caused an increased vertical activity in the Ex-SZT group in comparison with the two sedentary groups (vs Sed-veh, *p* = 0.018 and vs Sed-SZT, *p* = 0.011) (Figure 2b) (two-way ANOVA, significant main effect only of the exercise [*F*(1, 33) = 7.478, *p* = 0.01] on the vertical movements).

### 3.2 Passive-avoidance step-down test

**Figure 3:**
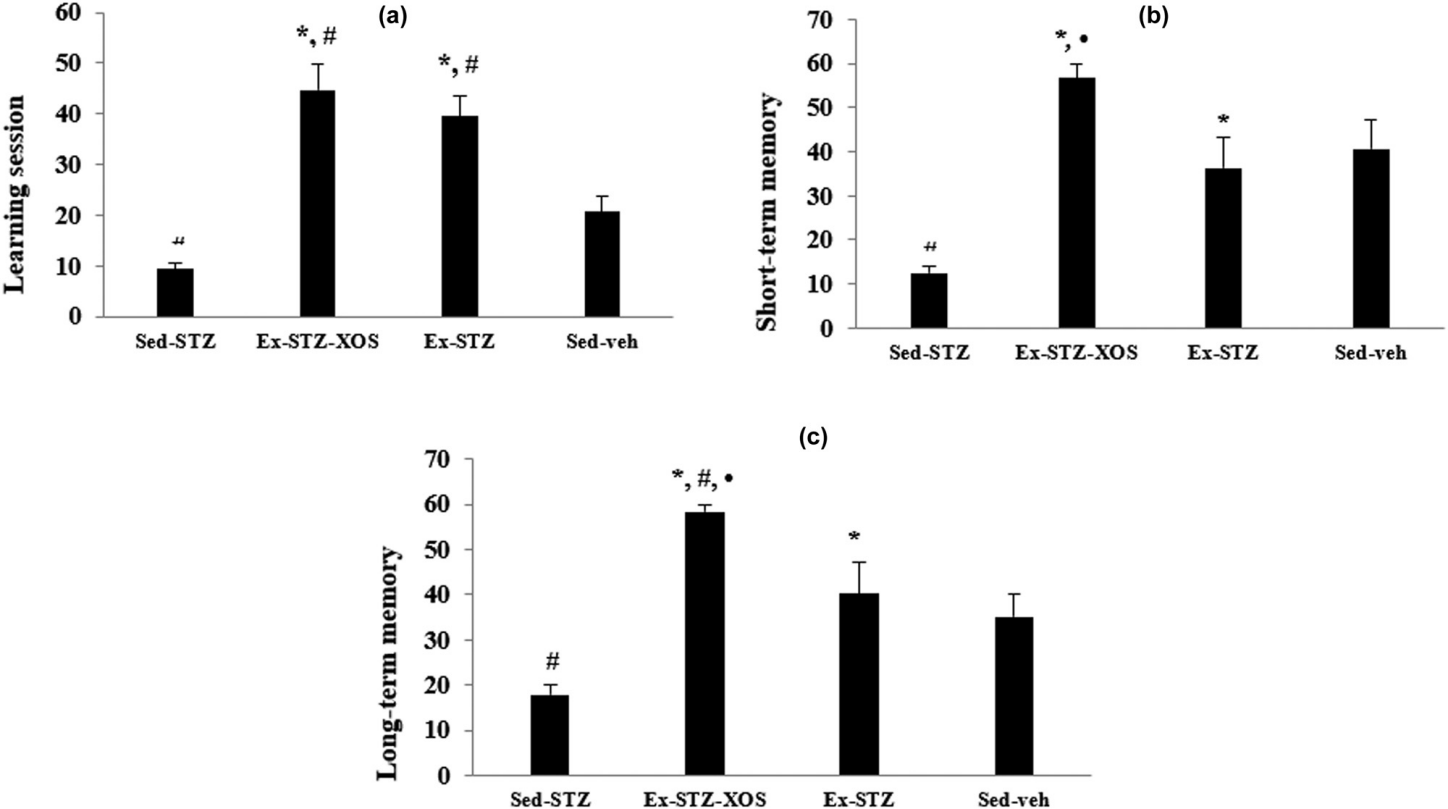
Effect of XOS and/or the aerobic training on the results from the passive-avoidance step-down test. (a) Learning session; (b) Short-term memory; (c) Long-term memory. ^*^Significant differences in comparison to Sed-STZ; ^#^significant differences in comparison to Sed-veh; and •significant differences in comparison to Ex-STZ.

The two-way ANOVA revealed significant effects of the diabetes [*F*(1, 33) = 4.469, *p* < 0.05] and exercise [*F*(1, 33) = 54.038, *p* < 0.001] on the learning session in the Step-down test. The *post hoc* test demonstrated that the Sed-SZT group had a shorter latency reaction than both the exercised groups (Ex-SZT and Ex-STZ-XOS, *p* < 0.001, resp.). The control group (Sed-veh) also had shorter time to spend on the platform compared to the Ex-SZT and Ex-STZ-XOS animals (*p* < 0.01) (Figure 3a).

In the test for the short-term memory traces, the *post hoc* test demonstrated that the Sed-SZT group decreased the latency time in comparison with the controls (*p* = 0.003) and both trained groups: Ex-SZT (*p* = 0.013) and Ex-STZ-XOS (*p* < 0.001). Furthermore, the exercised group with XOS supplementation spent more time on the platform compared to the Ex-SZT animals (*p* = 0.041) (Figure 3b) (two-way ANOVA, significant main effects of exercise [*F*(1, 33) = 24.089, *p* < 0.001] and diabetes [*F*(1, 33) = 12.310, *p* = 0.001] on the short-term memory traces).

During the long-term memory retention test, the analysis of variance demonstrated a significant decrease in the time spent on the platform for the Sed-SZT group compared to the other three groups: Sed-veh (*p* = 0.05), Ex-SZT (*p* = 0.007), and Ex-STZ-XOS (*p* < 0.001). The Ex-STZ-XOS animals also had better performance than the Sed-veh group (*p* = 0.005) and the Ex-STZ group (*p* = 0.035) (Figure 3c) (two-way ANOVA, significant main effects of exercise [*F*(1, 33) = 26.804, *p* < 0.001] and diabetes [*F*(1, 33) = 6.018, *p* = 0.02] on the long-term memory traces).

### 3.3 Elevated plus maze test

**Figure 4:**
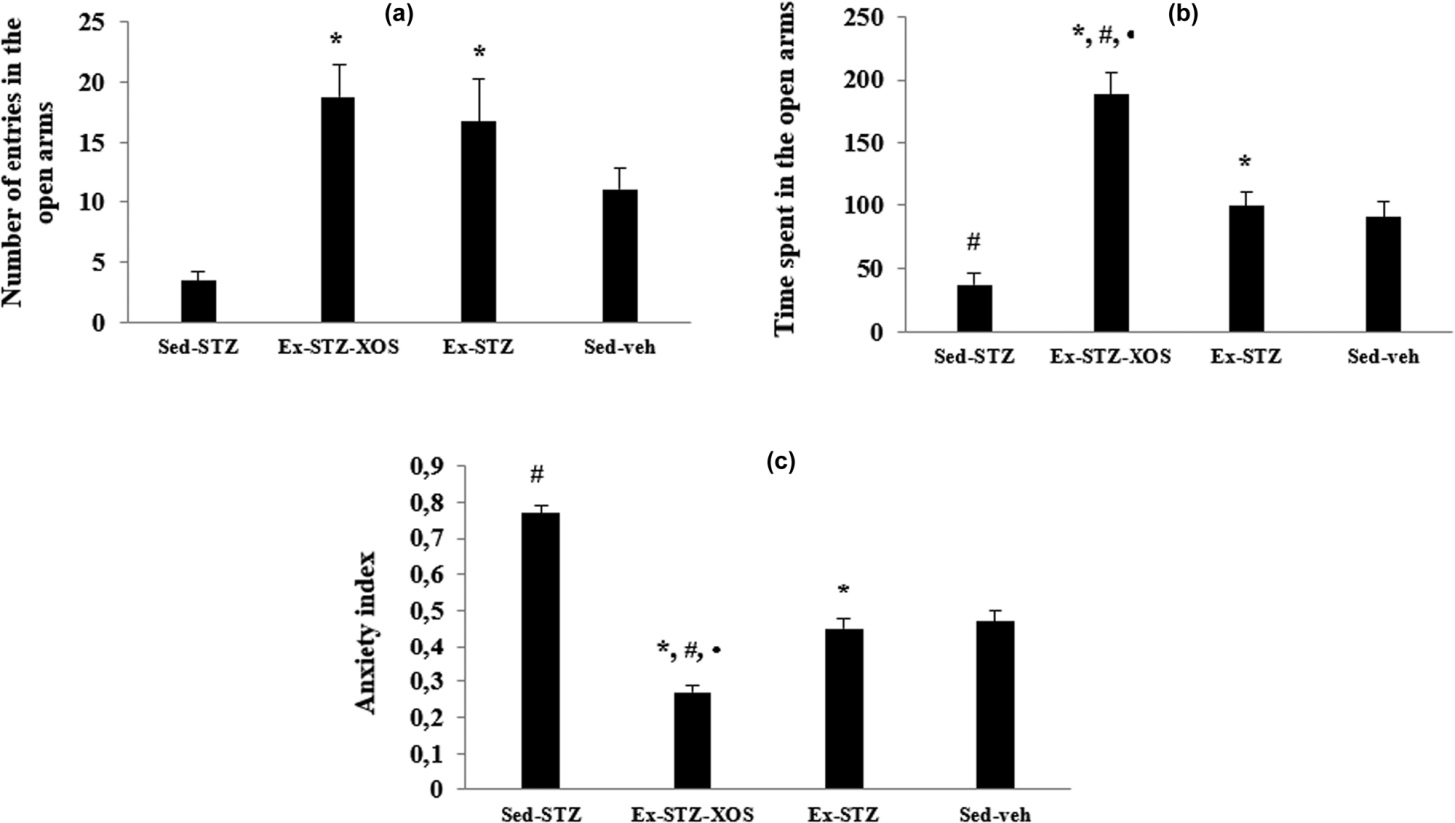
Effect of XOS and/or the aerobic training on the results from the elevated plus maze test. (a) Number of entries in the open arms; (b) Time spent in the open arms; (c) Anxiety index. ^*^Significant differences in comparison to Sed-STZ; ^#^significant differences in comparison to Sed-veh; and •significant differences in comparison to Ex-STZ.

The two-way ANOVA demonstrated significant main effects of the exercise [*F*(1, 33) = 24.281, *p* < 0.001] and diabetes [*F*(1, 33) = 5.060, *p* = 0.031] on the number of entries in the open arms. The *post hoc* test revealed that both exercised groups (Ex-STZ and Ex-STZ-XOS) had higher numbers of entries in the open arms in comparison with the Sed-SZT animals (*p* < 0.01, resp.) (Figure 4a) and longer time spent in the aversive area (Ex-STZ vs Sed-SZT animals, *p* = 0.008 and Ex-STZ-XOS vs Sed-STZ animals, *p* < 0.001). The Sed-STZ group also spent less time in the open arms compared to the control group (*p* = 0.028). Moreover, the Ex-STZ-XOS animals increased the time spent in the open arms in comparison with the controls and the Ex-SZT group (*p* < 0.001) (Figure 4b) (two-way ANOVA, significant main effects of exercise [*F*(1, 33) = 27.331, *p* < 0.001] and diabetes [*F*(1, 33) = 5.114, *p* = 0.03] on the time spent in the open arms).

The main effects of both diabetes [*F*(1, 33) = 29.167, *p* = 0.001] and exercise [*F*(1, 33) = 87.529, *p* < 0.001] were significant for the anxiety index. The *post hoc* test demonstrated that the Sed-SZT animals had higher anxiety index compared to Sed-veh animals (*p* < 0.001), what is more both the trained groups decreased the anxiety levels compared to the Sed-SZT animals (*p* < 0.001, resp.). The exercised group with XOS supplementation had even lower anxiety index compared to the Ex-SZT animals (*p* = 0.001) and the control group (*p* < 0.001) (Figure 4c).

### 3.4 Y-maze

**Figure 5:**
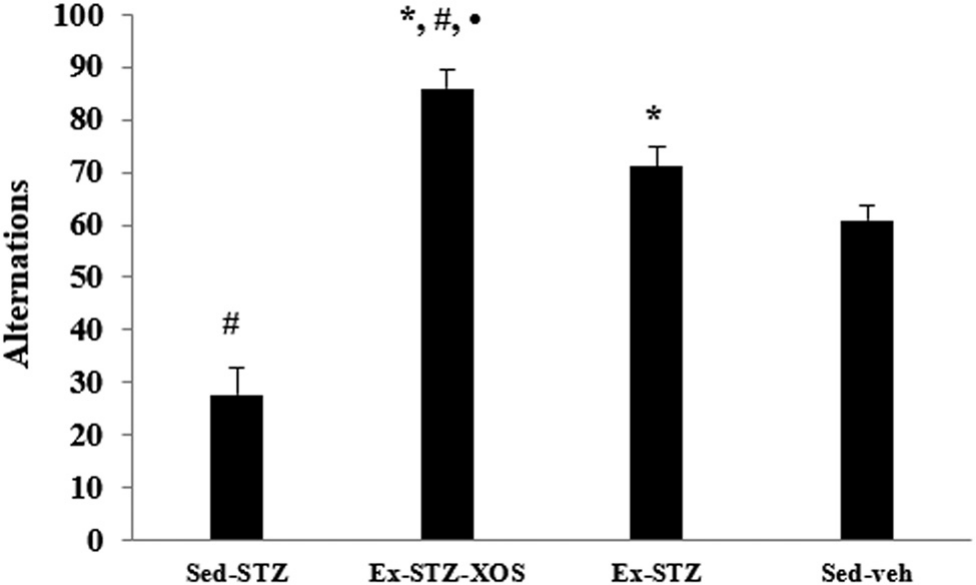
Effect of XOS and/or the aerobic training on the results from the Y-maze test. ^*^Significant differences in comparison to Sed-STZ; ^#^significant differences in comparison to Sed-veh; and •significant differences in comparison to Ex-STZ.

Percentages of alternations were greatly increased by both exercised groups when compared to the sedentary animals with T1DM (Ex-SZT vs Sed-SZT animals and Ex-STZ-XOS vs Sed-SZT animals, *p* < 0.001, resp.) The Sed-SZT animals also lowered them when compared to the Sed-veh group (*p* < 0.001). Furthermore, chronic supplementation with XOS additionally increased the percentages of alternations in comparison to the Ex-STZ group (*p* = 0.045) and the control group (*p* < 0.001) (Figure 5) {two-way ANOVA, significant main effects of the exercise [*F*(1, 33) = 97.975, *p* < 0.001] and diabetes [*F*(1, 33) = 31.387, *p* = 0.001] on the percentages of alternations}.

### 3.5 ORT

**Figure 6:**
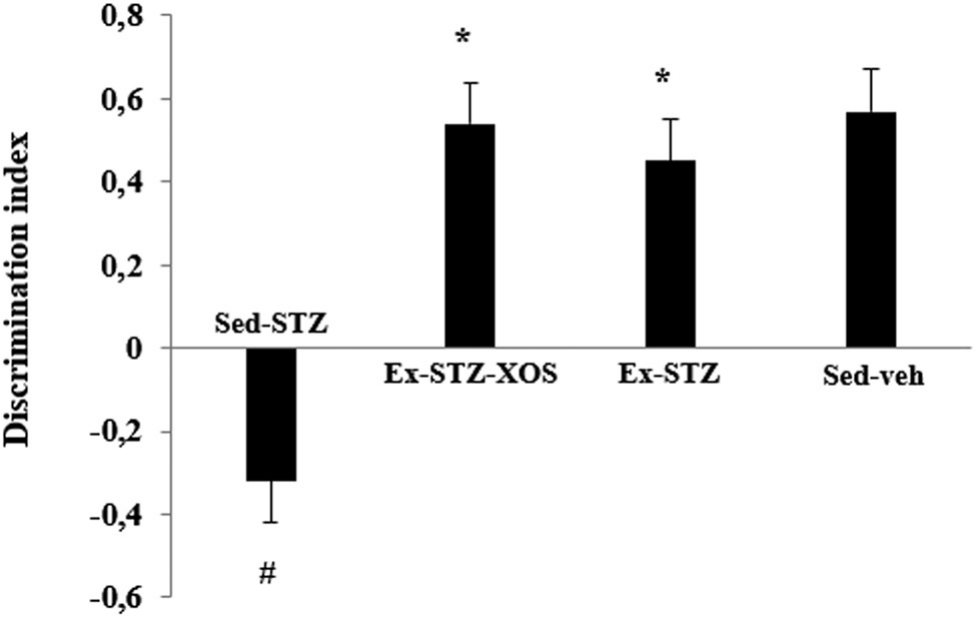
Effect of XOS and/or the aerobic training on the results from the ORT. ^*^Significant differences in comparison to Sed-STZ and ^#^significant differences in comparison to Sed-veh.

Two-way ANOVA revealed significant main effects of the exercise [*F*(1, 33) = 42.366, *p* < 0.001] and diabetes [*F*(1, 33) = 38.743, *p* = 0.001] on the discrimination index. The *post hoc* comparison indicated that both exercised groups (Ex-STZ and Ex-STZ-XOS) and the control group were characterized by an increased discrimination index when compared to the sedentary animals with diabetes (*p* < 0.001, resp.) (Figure 6).

### 3.6 Lipid profile

The obtained results regarding the studied parameters of the lipid profile of the four experimental animal groups are presented in Table 1.

**Table 1:** Descriptive statistics of the parameters from the lipid profile

Groups	TC (µmol/L)		TAG (µmol/L)		HDL (ng/mL)	
	Mean	SEM	Mean	SEM	Mean	SEM
(1) Sed-STZ	2.10	0.19	1.69^#^	0.02	6.83	0.57
(2) Ex-STZ-XOS	1.58	0.16	1.41^#^	0.03	6.84	0.88
(3) Ex-STZ	0.86^*,#^	0.19	1.39^#^	0.05	5.98	0.56
(4) Sed-veh	2.29	0.13	0.41	0.22	6.04	0.52

*Significant differences with *p* < 0.05 in comparison to Sed-STZ.

^#^Significant differences with *p* < 0.01 in comparison to Sed-veh.

Statistically significant differences in the concentration of the TC were found between the Ex-STZ group and the Sed-STZ group (*p* = 0.032), as well as between the Ex-STZ group and the Sed-veh group (*p* = 0.010).

The serum TAG levels of the Sed-veh group were found to be significantly lower than all other three groups: Sed-STZ (*p* = 0.000), Ex-STZ-XOS (*p* = 0.000), and Ex-XOS (*p* = 0.000).

### 3.7 Oxidative stress markers

The obtained results regarding the studied oxidative stress markers of the four experimental animal groups are presented in Table 2.

**Table 2:** Descriptive statistics of the oxidative stress markers

Groups	8-OH-dG (ng/mL)		FRAP (µmol/L)		MDA (ng/mL)	
	Mean	SEM	Mean	SEM	Mean	SEM
(1) Sed-STZ	29.92	11.79	239.75	14.61	87.23	21.94
(2) Ex-STZ-XOS	26.21	5.24	245.80	15.61	39.51^*^	17.34
(3) Ex-STZ	33.05	8.91	219.67	10.37	122.89	28.32
(4) Sed-veh	19.17	2.17	250.17	8.42	73.11	22.66

*Significant differences with *p* = 0.034 in comparison to Ex-STZ.

Regarding the markers of the oxidative stress, we found a statistically significant difference in the concentration of MDA between the Ex-STZ group and the Ex-STZ-XOS group (*p* = 0.034), being lower in the Ex-STZ-XOS group.

## 4 Discussion

The aim of our experiment was to determine whether the prebiotic XOS could beneficially affect the cognition, lipid profile, and oxidative stress status of type 1 diabetic rats and aid the effect of the aerobic training. Although prebiotics have lately been widely studied, there is still very limited information on the effects of XOS and especially their influence on cognition.

In our study, we found that rats with STZ-induced T1DM have cognitive deficits accompanied with increased anxiety levels. Aerobic training managed to ameliorate the cognitive dysfunction through increasing the passive learning and short-term memory, while on the formation of long-term memory traces both exercise and XOS supplementation exert a positive effect. Moreover, spatial memory deficits observed in the diabetic rats in the Y-maze test were overcome by endurance training and prebiotic treatment and, thus, their spatial memory performance was improved. In the object recognition memory task, the control animals and both groups with aerobic training with/without XOS supplementation spent more time exploring the novel object than the familiar one and thus showed improved memory consolidation and retrieval. In contrast, rats with induced T1DM spent more time exploring the familiar object, which is a manifestation of impaired recognition memory due to the diabetes.

Recent data have shown the link between gut microbiota, cognitive function, and microglia where long-term consumption of XOS ameliorated the activated microglia and restored the cognition in diet-induced obese rats [30]. In the present study, prebiotic administration and aerobic training managed to reduce the anxiety levels in the elevated plus maze test through increasing the entries in the open arms and the time spent in them. Our results complete other experimental data revealing that supplementation with other prebiotics such as galacto- and fructooligosaccharides could decrease the anxiety-like behavior under healthy or stressful conditions or after lipopolysaccharide injection in different rodents [31,32]. Moreover, previous data have shown that prebiotic treatment and exercise enhance the growth and activity of stress-protective bacteria, such as *Lactobacillus*, *Bifidobacteria*, and butyrate-producing bacteria [33], which could in turn decrease the release of pro-inflammatory cytokines, therefore protecting the hippocampus from immune changes [34].

It is well known that aerobic training as a non-pharmacological approach has positive effects on cognitive functions. Regular physical exercise improves brain function by increasing the gene expression of the brain-derived neurotrophic factor, neurogenesis, synaptic plasticity, and hippocampal cell density, which in turn enhances hippocampal-dependent learning and memory [35,36]. Recent data have shown that aerobic training can also induce an anxiolytic effect in diabetic rats, which is documented by an increase in the total entries and time spent in the central region in the OF test [37]. Moreover, a number of studies have shown similar effects of aerobic training, which decreases cortisol concentration, normalizes serotonergic and noradrenergic concentration and corrects the release of corticotropin-releasing hormone, and in turn modulates the responsiveness to anxiety and stress [38].

The biochemical relationship between TAG and cognitive/mood disorders is not well elucidated. Our findings show that high levels of TAG are likely a major cause for the observed cognitive disturbances in our model of STZ-induced T1DM. These results are in compliance with other studies reporting that TAG lead to working memory errors and spatial memory deficits in models of diet-induced obesity and the effect is reversed pharmacologically by the reduction of their levels [7]. There are data that TAG can impair the maintenance of *N*-methyl-d-aspartate-dependent hippocampal long-term synaptic potentiation, which is important for the synaptic plasticity and therefore for learning and memory [7,39]. In addition, recent data have shown that TAG can cross the blood–brain barrier (BBB) in experimental conditions with intravenous injection of radioactive triolein and can induce central receptor resistance to leptin and insulin and thus can additionally aggravate the cognitive dysfunction [40].

Another factor observed in our study that can have detrimental effects on learning and memory is the high levels of TC. Increasing number of experimental and human data support the hypothesis that high cholesterol and saturated fat intake is strongly correlated with poor cognitive performance not only in the aged population but in school children as well [41–43]. In rodents with experimental models of obesity, impairment in the hippocampal-dependent learning and memory has also been detected [7,44,45]. There are data that cholesterol imbalance induces changes in brain pathology through modifying the electrophysiological properties of the neurons especially in the hippocampus [46]. Previous studies have demonstrated that high-fat diet consumption triggers microglia activation, leading to hippocampal dysplasticity and activated cell apoptosis resulting in cognitive decline [47,48]. Moreover, BBB permeability in such models is also increased and thus brain exposure to different pro-inflammatory cytokines, such as IL-1β, IL-6, and TNF-α, is increased [49]. A potential mechanism that could be involved in the cholesterol-induced cognitive impairment is the greater propensity to atherosclerosis in transgenic mice after high-fat diet [50].

Regarding the lipid profile, the obtained results show that XOS supplementation and/or aerobic training exert a beneficial effect on the serum concentrations of TAG and TC. Our results are in accordance with those reported by Rios et al., for the effect of prebiotic fiber and aerobic exercise, leading to improvement of the serum lipid profile of rats fed with a high-fat/high-sucrose diet [51]. We found that the healthy control group has lower TAG serum concentration compared to all diabetic animals. Our results show that aerobic training independently as well as in combination with XOS could lead to a decrease in the TAG levels, although the difference is not statistically significant. Some studies suggest that this positive effect could be due to increased serum lipid consumption by the skeletal muscles utilizing the fatty acids in the TAG of very-low-density lipoproteins (VLDL) or chylomicrons as fuel in aerobic exercises and by stimulation of the skeletal lipoprotein lipase (LPL) activity and mRNA synthesis [52]. Another mechanism for decreasing the TAG levels in endurance trained experimental animals might be the inhibition of the apoC3 protein, as it was suggested by Wang et al. in patients with coronary heart disease [53]. It is well recognized that apoC_3_ is involved in plasma TAG metabolism by inhibiting LPL and hepatic lipase, the enzymes that hydrolyze TAG in lipoproteins. Thus, apoC_3_ reduces the uptake of VLDL and chylomicron remnants by hepatocytes and maintains high plasma TAG levels [54]. Several studies report that XOS and other oligosaccharides could lower the serum TAG concentrations through increasing the production of SCFAs in the large intestine [55–57], where they serve as major signaling molecules and act as ligands for several receptors, such as free fatty acid receptor 2 (FFAR2) and FFAR3 [58]. The activation of these receptors leads to an increased release of some anorectic peptides, such as glucagon-like peptide-1 (GLP-1) and peptide YY, which results in decreased levels of TAG and TC [59,60]. Dietary fibers are also found to stimulate leptin production and thus participate in the regulation of the energy homeostasis [61].

According to the obtained results aerobic training managed to significantly lower the serum TC concentration in the diabetic rats. XOS supplementation combined with aerobic training also led to a decrease in the TC levels; however, the effect was not so pronounced. A possible mechanism by which dietary oligosaccharides could lead to a reduction of the serum cholesterol levels includes reduced cholesterol absorption and increased excretion in the feces [57,62].

Emerging evidence suggests that oxidative stress plays a pivotal role in behavioral and mood alterations and can be influenced by prebiotic treatment [63]. Prebiotic supplementation is found to decrease oxidative stress and microglia activation leading to re-established hippocampal plasticity accompanied with improved mood performance [30]. Our results show that XOS supplementation leads to a significant decrease in the serum MDA concentration. The exact mechanism by which prebiotics alleviate oxidative stress have not been discovered yet; however, some authors suggest that this effect could be due to an increased expression and activity of antioxidant enzymes and direct neutralization of oxidants [64].

## 5 Conclusions

This study indicates that XOS supplementation independently as well as in combination with aerobic training confers several beneficial effects in type 1 STZ-induced diabetic rats regarding the dyslipidemia and oxidative stress – conditions related to cognitive deficits. The reduced hypertriglyceridemia and hypercholesterolemia as well as amelioration of the oxidative stress status could have in turn contributed to the alleviation of the cognitive deficits accomplished by the XOS and/or aerobic training. Further investigation will be needed for the mechanism of these effects to be studied.

### 5.1 Limitations and future implications

A main limitation of this study was the small sample size, which is typical of animal studies. A small number of animals in an experimental group are a requirement of the ethical guidelines for the use of animals in research. The death of some of the animals during the experiment also impacted the sample size. As we aimed to investigate the beneficial effects of functional oligosaccharides and aerobic training on multiple levels and from different points of view, a large dataset was generated, which led to its distribution amongst several articles. This was yet another limitation of the study.

Oligosaccharides have gained popularity during the recent years, which has to do with their multiple beneficial effects on human health. Since data regarding their impact on cognition and especially the one of XOS are still very limited, our study offers some insight on the matter. This raises some opportunities for future research and for us to refine and elaborate our findings.

## Abbreviations

ANOVA: analysis of variance
BAFS: Bulgarian Agency for Food Safety
BBB: blood–brain barrier
DI: discrimination index
DM: diabetes mellitus
DP: degree of polymerization
ELISA: enzyme-linked immunosorbent assay
FFAR2: free fatty acid receptor 2
FFAR3: free fatty acid receptor 3
FRAP: ferric-reducing ability of plasma
GLP-1: glucagon like peptide-1
HDL: high-density lipoproteins
LPL: lipoprotein lipase
MDA: malondialdehyde
OF: open field
ORT: object recognition test
ROS: reactive oxygen species
SCFAs: short-chain fatty acids
SEM: standard error of mean
STZ: streptozotocin
TAG: triacylglycerols
TC: total cholesterol
T1DM: type 1 diabetes mellitus
TPTZ: 2,4,6-tripyridyl-*s*-triazine
VLDL: very-low-density lipoproteins
XOS: xylooligosaccharides
8-OH-dG: 8-hydroxy-2-deoxyguanosine

## References

[1] Lamichhane S, Ahonen L, Dyrlund TS, Siljander H, Hyöty H, Ilonen J, et al. A longitudinal plasma lipidomics dataset from children who developed islet autoimmunity and type 1 diabetes. Sci Data. 2018 Nov 13;5:180250. 10.1038/sdata.2018.250 PMC6233478 30422126

[2] Rewers M, Ludvigsson J. Environmental risk factors for type 1 diabetes. Lancet. 2016 Jun;387(10035):2340–8. 10.1016/S0140-6736(16)30507-4 PMC5571740 27302273

[3] Vaarala O. Gut microbiota and type 1 diabetes. Rev Diabet Stud. 2012;9(4):251–9. 10.1900/RDS.2012.9.251 PMC3740694 23804264

[4] Etgen T, Sander D, Bickel H, Sander K, Förstl H. Cognitive decline: the relevance of diabetes, hyperlipidaemia and hypertension. Br J Diabetes Vasc Dis. 2010 May;10(3):115–22.

[5] Shalimova A, Graff B, Gąsecki D, Wolf J, Sabisz A, Szurowska E, et al. Cognitive dysfunction in type 1 diabetes mellitus. J Clin Endocrinol Metab. 2019 Jan 18;104(6):2239–49. 10.1210/jc.2018-01315 30657922

[6] Zabeen B, Balsa A, Islam N, Parveen M, Nahar J, Azad K. Lipid profile in relation to glycemic control in Type 1 diabetes children and adolescents in Bangladesh. Indian J Endocrinol Metab. 2018;22(1):89. 10.4103/ijem.IJEM_217_17 PMC5838919 29535944

[7] Farr SA, Yamada KA, Butterfield DA, Abdul HM, Xu L, Miller NE, et al. Obesity and hypertriglyceridemia produce cognitive impairment. Endocrinology. 2008 Feb 14;149(5):2628–36. 10.1210/en.2007-1722 PMC2329289 18276751

[8] Ullrich C, Pirchl M, Humpel C. Hypercholesterolemia in rats impairs the cholinergic system and leads to memory deficits. Mol Cell Neurosci. 2010 Dec;45(4):408–17. 10.1016/j.mcn.2010.08.001 PMC2977849 20696249

[9] Muriach M, Flores-Bellver M, Romero FJ, Barcia JM. Diabetes and the brain: Oxidative stress, inflammation, and autophagy. Oxid Med Cell Longev. 2014;2014:1–9. 10.1155/2014/102158 PMC4158559 25215171

[10] Heijtz RD, Wang S, Anuar F, Qian Y, Bjorkholm B, Samuelsson A, et al. Normal gut microbiota modulates brain development and behavior. Proc Natl Acad Sci. 2011 Jan 31;108(7):3047–52. 10.1073/pnas.1010529108 PMC3041077 21282636

[11] Desbonnet L, Clarke G, Shanahan F, Dinan TG, Cryan JF. Microbiota is essential for social development in the mouse. Mol Psychiatry. 2013 May 21;19(2):146–8. 10.1038/mp.2013.65 PMC3903109 23689536

[12] Fung TC, Olson CA, Hsiao EY. Interactions between the microbiota, immune and nervous systems in health and disease. Nat Neurosci. 2017 Jan 16;20(2):145–55. 10.1038/nn.4476 PMC6960010 28092661

[13] Smith PA. The tantalizing links between gut microbes and the brain. Nature. 2015 Oct 14;526(7573):312–4. 10.1038/526312a 26469024

[14] Tremlett H, Bauer KC, Appel-Cresswell S, Finlay BB, Waubant E. The gut microbiome in human neurological disease: A review. Ann Neurol. 2017 Mar;81(3):369–82. 10.1002/ana.24901 28220542

[15] Mishra S, Wang S, Nagpal R, Miller B, Singh R, Taraphder S, et al. Probiotics and prebiotics for the amelioration of type 1 diabetes: Present and future perspectives. Microorganisms. 2019 Mar 2;7(3):67. 10.3390/microorganisms7030067 PMC6463158 30832381

[16] Jordan DB, Wagschal K. Properties and applications of microbial beta-D-xylosidases featuring the catalytically efficient enzyme from Selenomonas ruminantium. Appl Microbiol Biotechnol. 2010 May 1;86(6):1647–58. 10.1007/s00253-010-2538-y 20352422

[17] Sheu WH, Lee IT, Chen W, Chan YC. Effects of xylooligosaccharides in type 2 diabetes mellitus. J Nutr Sci Vitaminol. 2008;54(5):396–401. 10.3177/jnsv.54.396 19001772

[18] Yang J, Summanen PH, Henning SM, Hsu M, Lam H, Huang J, et al. Xylooligosaccharide supplementation alters gut bacteria in both healthy and prediabetic adults: A pilot study. Front Physiol. 2015 Aug 7;6:216. 10.3389/fphys.2015.00216 PMC4528259 26300782

[19] Mika A, Gaffney M, Roller R, Hills A, Bouchet CA, Hulen KA, et al. Feeding the developing brain: Juvenile rats fed diet rich in prebiotics and bioactive milk fractions exhibit reduced anxiety-related behavior and modified gene expression in emotion circuits. Neurosci Lett. 2018 Jun;677:103–9. 10.1016/j.neulet.2018.01.052 29409860

[20] Zhao J, Tian F, Yan S, Zhai Q, Zhang H, Chen W. Lactobacillus plantarum CCFM10 alleviating oxidative stress and restoring the gut microbiota in d-galactose-induced aging mice. Food Funct. 2018;9(2):917–24. 10.1039/c7fo01574g 29313548

[21] Krishna G. Oral supplements of combined fructo- and xylo-oligosaccharides during perinatal period significantly offsets acrylamide-induced oxidative impairments and neurotoxicity in rats. J Physiol Pharmacol: An Off J Pol Physiol Soc. 2018 Oct 1;69(5):801–14. 10.26402/jpp.2018.5.14 30683831

[22] Yuing T, Lizana PA, Berral FJ. Effects of physical training in patients with type 2 diabetes mellitus: A systematic review. Rev Med Chil. 2019 Apr 1;147(4):480–9. 10.4067/S0034-98872019000400480 31344211

[23] Jenkins DW, Jenks A. Exercise and diabetes: A narrative review. J Foot Ankle Surg. 2017;56(5):968–74. 10.1053/j.jfas.2017.06.019 28842107

[24] Nery C, De Moraes SR, Novaes KA, Bezerra MA, Silveira PV, Lemos A. Effectiveness of resistance exercise compared to aerobic exercise without insulin therapy in patients with type 2 diabetes mellitus: A meta-analysis. Braz J Phys Ther. 2017 Nov;21(6):400–15. 10.1016/j.bjpt.2017.06.004 PMC5693273 28728958

[25] Reid RD, Tulloch HE, Sigal RJ, Kenny GP, Fortier M, McDonnell L, et al. Effects of aerobic exercise, resistance exercise or both, on patient-reported health status and well-being in type 2 diabetes mellitus: a randomised trial. Diabetologia. 2009 Dec 13;53(4):632–40. 10.1007/s00125-009-1631-1 20012857

[26] Furman BL. Streptozotocin-induced diabetic models in mice and rats. Curr Protoc Pharmacol. 2015 Sep 1;5(47):1–20. 10.1002/0471141755.ph0547s70 26331889

[27] Akbarzadeh A, Norouzian D, Mehrabi MR, Jamshidi SH, Farhangi A, Verdi AA, et al. Induction of diabetes by Streptozotocin in rats. Indian J Clin Biochem. 2007 Sep;22(2):60–4. 10.1007/BF02913315 PMC3453807 23105684

[28] Manchado-Gobatto FB, Gobatto CA, Contarteze RVL, Papoti M, De Mello MAR. Maximal lactate steady state in running rats. J Exerc Physiol Online. 2005;8:29–35.

[29] Benzie IFF, Strain JJ. The ferric reducing ability of plasma (FRAP) as a measure of “Antioxidant Power”: The FRAP assay. Anal Biochem. 1996 Jul;239(1):70–6. 10.1006/abio.1996.0292 8660627

[30] Chunchai T, Thunapong W, Yasom S, Wanchai K, Eaimworawuthikul S, Metzler G, et al. Decreased microglial activation through gut–brain axis by prebiotics, probiotics, or synbiotics effectively restored cognitive function in obese-insulin resistant rats. J Neuroinflammation. 2018 Jan 9;15:11. 10.1186/s12974-018-1055-2 PMC5761137 29316965

[31] Savignac HM, Couch Y, Stratford M, Bannerman DM, Tzortzis G, Anthony DC, et al. Prebiotic administration normalizes lipopolysaccharide (LPS)-induced anxiety and cortical 5-HT2A receptor and IL1-β levels in male mice. Brain Behav Immun. 2016 Feb;52:120–31. 10.1016/j.bbi.2015.10.007 PMC4927692 26476141

[32] Burokas A, Arboleya S, Moloney RD, Peterson VL, Murphy K, Clarke G, et al. Targeting the microbiota-gut–brain axis: Prebiotics have anxiolytic and antidepressant-like effects and reverse the impact of chronic stress in mice. Biol Psychiatry. 2017 Oct;82(7):472–87. 10.1016/j.biopsych.2016.12.031 28242013

[33] Mika A, Rumian N, Loughridge AB, Fleshner M. Exercise and prebiotics produce stress resistance. Int Rev Neurobiol. 2016;131:165–91. 10.1016/bs.irn.2016.08.004 27793217

[34] Li N, Wang Q, Wang Y, Sun A, Lin Y, Jin Y, et al. Oral probiotics ameliorate the behavioral deficits induced by chronic mild stress in mice via the gut microbiota-inflammation axis. Front Behav Neurosci. 2018 Nov 6;12:266. 10.3389/fnbeh.2018.00266 PMC6232506 30459574

[35] Krüger K, Bredehöft J, Mooren FC, Rummel C. Different effects of strength and endurance exercise training on COX-2 and mPGES expression in mouse brain are independent of peripheral inflammation. J Appl Physiol. 2016 Jun 9;121(1):248–54. 10.1152/japplphysiol.00284.2016 27283912

[36] Shishmanova-Doseva M, Georgieva K, Koeva Y, Terzieva D, Peychev L. Enhancing effect of aerobic training on learning and memory performance in rats after long-term treatment with Lacosamide via BDNF-TrkB signaling pathway. Behavioural Brain Res. 2019 Sep;370:111963. 10.1016/j.bbr.2019.111963 31116960

[37] Caliskan H, Akat F, Tatar Y, Zaloglu N, Dursun AD, Bastug M, et al. Effects of exercise training on anxiety in diabetic rats. Behavioural Brain Res. 2019 Dec;376:112084. 10.1016/j.bbr.2019.112084 31356829

[38] Brondino N, Rocchetti M, Fusar-Poli L, Codrons E, Correale L, Vandoni M, et al. A systematic review of cognitive effects of exercise in depression. Acta Psychiat Scand. 2017 Jan 22;135(4):285–95. 10.1111/acps.12690 28110494

[39] Malenka RC, Bear MF. LTP and LTD: An embarrassment of riches. Neuron. 2004;44(1):5–21. 10.1016/j.neuron.2004.09.012 15450156

[40] Banks WA, Farr SA, Salameh TS, Niehoff ML, Rhea EM, Morley JE, et al. Triglycerides cross the blood–brain barrier and induce central leptin and insulin receptor resistance. Int J Obes. 2017 Oct 9;42(3):391–7. 10.1038/ijo.2017.231 PMC5880581 28990588

[41] Teunissen C. Serum cholesterol, precursors and metabolites and cognitive performance in an aging population. Neurobiol Aging. 2003 Feb;24(1):147–55. 10.1016/s0197-4580(02)00061-1 12493560

[42] Zhang J, Hebert JR, Muldoon MF. Dietary fat intake is associated with psychosocial and cognitive functioning of school-aged children in the united states. J Nutr. 2005 Aug 1;135(8):1967–73. 10.1093/jn/135.8.1967 16046724

[43] Nilsson A, Radeborg K, Salo I, Björck I. Effects of supplementation with n-3 polyunsaturated fatty acids on cognitive performance and cardiometabolic risk markers in healthy 51 to 72 years old subjects: A randomized controlled cross-over study. Nutr J. 2012 Nov 22;11(1):99. 10.1186/1475-2891-11-99 PMC3564898 23173831

[44] Granholm A-C, Bimonte-Nelson HA, Moore AB, Nelson ME, Freeman LR, Sambamurti K. Effects of a saturated fat and high cholesterol diet on memory and hippocampal morphology in the middle-aged rat. J Alzheimers Dis. 2008 Jun 4;14(2):133–45. 10.3233/jad-2008-14202 PMC2670571 18560126

[45] Jurdak N, Kanarek RB. Sucrose-induced obesity impairs novel object recognition learning in young rats. Physiol & Behav. 2009 Jan 8;96(1):1–5. 10.1016/j.physbeh.2008.07.023 18718844

[46] Wang D, Schreurs BG. Dietary cholesterol modulates the excitability of rabbit hippocampal CA1 pyramidal neurons. Neurosci Lett. 2010 Aug;479(3):327–31. 10.1016/j.neulet.2010.05.090 PMC3000631 20639007

[47] Hao S, Dey A, Yu X, Stranahan AM. Dietary obesity reversibly induces synaptic stripping by microglia and impairs hippocampal plasticity. Brain Behav Immun. 2016 Jan;51:230–9. 10.1016/j.bbi.2015.08.023 PMC4679537 26336035

[48] Chunchai T, Samniang B, Sripetchwandee J, Pintana H, Pongkan W, Kumfu S, et al. Vagus nerve stimulation exerts the neuroprotective effects in obese-insulin resistant rats, leading to the improvement of cognitive function. Sci Rep. 2016 May 26;6(1):26866. 10.1038/srep26866 PMC4880928 27226157

[49] Freeman LR, Haley-Zitlin V, Rosenberger DS, Granholm A-C. Damaging effects of a high-fat diet to the brain and cognition: A review of proposed mechanisms. Nutritional Neurosci. 2013 Nov 26;17(6):241–51. 10.1179/1476830513Y.0000000092 PMC4074256 24192577

[50] Li L, Cao D, Garber DW, Kim H, Fukuchi K. Association of aortic atherosclerosis with cerebral β-amyloidosis and learning deficits in a mouse model of Alzheimer’s disease. Am J Pathol. 2003 Dec;163(6):2155–64. 10.1016/s0002-9440(10)63572-9 PMC1892402 14633589

[51] Rios JL, Bomhof MR, Reimer RA, Hart DA, Collins KH, Herzog W. Protective effect of prebiotic and exercise intervention on knee health in a rat model of diet-induced obesity. Sci Rep. 2019 Mar 7;9(1):3893. 10.1038/s41598-019-40601-x PMC6405910 30846801

[52] Kersten S. Physiological regulation of lipoprotein lipase. Biochim Biophys Acta Mol Cell Biol Lipids. 2014 Jul;1841(7):919–33. 10.1016/j.bbalip.2014.03.013 24721265

[53] Wang Y, Shen L, Xu D. Aerobic exercise reduces triglycerides by targeting apolipoprotein C3 in patients with coronary heart disease. Clin Cardiology. 2018 Dec 21;42(1):56–61. 10.1002/clc.23104 PMC6436502 30511426

[54] Norata GD, Tsimikas S, Pirillo A, Catapano AL. Apolipoprotein C-III: From Pathophysiology to Pharmacology. Trends Pharmacol Sci. 2015 Oct;36(10):675–87. 10.1016/j.tips.2015.07.001 26435212

[55] Kok N, Roberfroid M, Robert A, Delzenne N. Involvement of lipogenesis in the lower VLDL secretion induced by oligofructose in rats. Br J Nutr. 1996 Dec;76(6):881–90. 10.1079/bjn19960094 9014656

[56] Beylot M. Effects of inulin-type fructans on lipid metabolism in man and in animal models. Br J Nutr. 2005 Apr 1;93(S1):S163–8. 10.1079/bjn20041339 15877890

[57] Gobinath D, Madhu AN, Prashant G, Srinivasan K, Prapulla SG. Beneficial effect of xylo-oligosaccharides and fructo-oligosaccharides in streptozotocin-induced diabetic rats. Br J Nutr. 2010 Feb 26;104(1):40–7. 10.1017/S0007114510000243 20187988

[58] De Vadder F, Kovatcheva-Datchary P, Goncalves D, Vinera J, Zitoun C, Duchampt A, et al. Microbiota-generated metabolites promote metabolic benefits via gut–brain neural circuits. Cell. 2014 Jan;156(1–2):84–96. 10.1016/j.cell.2013.12.016 24412651

[59] Tolhurst G, Heffron H, Lam YS, Parker HE, Habib AM, Diakogiannaki E, et al. Short-chain fatty acids stimulate glucagon-like peptide-1 secretion via the G-protein-coupled receptor FFAR2. Diabetes. 2011 Dec 21;61(2):364–71. 10.2337/db11-1019 PMC3266401 22190648

[60] Grenier E, Garofalo C, Delvin E, Levy E. Modulatory role of PYY in transport and metabolism of cholesterol in intestinal epithelial cells. PLoS ONE. 2012 Jul 23;7(7):e40992. 10.1371/journal.pone.0040992 PMC3402548 22844422

[61] Samuel BS, Shaito A, Motoike T, Rey FE, Backhed F, Manchester JK, et al. Effects of the gut microbiota on host adiposity are modulated by the short-chain fatty-acid binding G protein-coupled receptor, Gpr41. Proc Natl Acad Sci. 2008 Oct 17;105(43):16767–72. 10.1073/pnas.0808567105 PMC2569967 18931303

[62] Van Bennekum AM, Nguyen DV, Schulthess G, Hauser H, Phillips MC. Mechanisms of cholesterol-lowering effects of dietary insoluble fibres: relationships with intestinal and hepatic cholesterol parameters. Br J Nutr. 2005 Sep;94(3):331–7. 10.1079/bjn20051498 16176602

[63] Treviño S, Aguilar-Alonso P, Flores Hernandez JA, Brambila E, Guevara J, Flores G, et al. A high calorie diet causes memory loss, metabolic syndrome and oxidative stress into hippocampus and temporal cortex of rats. Synapse. 2015 Jun 30;69(9):421–33. 10.1002/syn.21832 26073877

[64] Javadi L, Khoshbaten M, Safaiyan A, Ghavami M, Mesgari Abbasi M, Pourghassem Gargari B. Pro- and prebiotic effects on oxidative stress and inflammatory markers in non-alcoholic fatty liver disease. Asia Pac J Clin Nutr. 2018 Sep;27(5):1031–9. 10.6133/apjcn.042018.05 30272851

